# Racial variations in processes of care for patients with community-acquired pneumonia

**DOI:** 10.1186/1472-6963-4-20

**Published:** 2004-08-10

**Authors:** Eric M Mortensen, John Cornell, Jeff Whittle

**Affiliations:** 1 VERDICT and Division of General Internal Medicine, Audie L. Murphy VA Hospital and University of Texas Health Science Center, San Antonio, USA; 2 Kansas City VA Medical Center and Division of General Medicine and Geriatrics, University of Kansas School of Medicine (JW), USA

## Abstract

**Background:**

Patients hospitalized with community acquired pneumonia (CAP) have a substantial risk of death, but there is evidence that adherence to certain processes of care, including antibiotic administration within 8 hours, can decrease this risk. Although national mortality data shows blacks have a substantially increased odds of death due to pneumonia as compared to whites previous studies of short-term mortality have found decreased mortality for blacks. Therefore we examined pneumonia-related processes of care and short-term mortality in a population of patients hospitalized with CAP.

**Methods:**

We reviewed the records of all identified Medicare beneficiaries hospitalized for pneumonia between 10/1/1998 and 9/30/1999 at one of 101 Pennsylvania hospitals, and randomly selected 60 patients at each hospital for inclusion. We reviewed the medical records to gather process measures of quality, pneumonia severity and demographics. We used Medicare administrative data to identify 30-day mortality. Because only a small proportion of the study population was black, we included all 240 black patients and randomly selected 720 white patients matched on age and gender. We performed a resampling of the white patients 10 times.

**Results:**

Males were 43% of the cohort, and the median age was 76 years. After controlling for potential confounders, blacks were less likely to receive antibiotics within 8 hours (odds ratio with 95% confidence interval 0.6, 0.4–0.97), but were as likely as whites to have blood cultures obtained prior to receiving antibiotics (0.7, 0.3–1.5), to have oxygenation assessed within 24 hours of presentation (1.6, 0.9–3.0), and to receive guideline concordant antibiotics (OR 0.9, 0.6–1.7). Black patients had a trend towards decreased 30-day mortality (0.4, 0.2 to 1.0).

**Conclusion:**

Although blacks were less likely to receive optimal care, our findings are consistent with other studies that suggest better risk-adjusted survival among blacks than among whites. Further study is needed to determine why this is the case.

## Background

Pneumonia (with influenza) is the leading infectious disease cause of death in the United States and the sixth leading cause of death overall [1] According to national mortality data from the CDC blacks suffer disproportionately from this disease, with blacks having a higher incidence of pneumonia and a 1.4 times higher age-adjusted odds of death from pneumonia as compared to non-Hispanic whites [1-4] In contrast several studies of racial variations in short-term mortality have demonstrated that black patients hospitalized with pneumonia are less likely to die in the hospital than whites. [5,6]

As part of the Pneumonia Medical Quality Improvement Study (MQIS) a national expert panel established a set of process of care measures for patients hospitalized with CAP [7] Several studies have demonstrated that some of these processes of care, especially prompt antibiotic administration within 4 or 8 hours of presentation, is associated with decreased mortality for inpatients with CAP [7-9] Although several studies have demonstrated significant racial differences in pneumonia care no one has examined whether there are racial variations in all of these explicit processes of care for patients with CAP. [5,6,10-13]

The aims of this paper are to 1) examine whether there are significant racial differences in the processes of care that have been associated with mortality for patients hospitalized with CAP, and 2) to examine the relative risks of death within 30-days for blacks versus whites.

## Methods

### Study patients

KePRO, the Medicare Peer Review Organization for Pennsylvania, obtained these data as part of the Pneumonia MQIS project, whose goals is to assess and improve the quality of care for Medicare patients hospitalized with CAP.

The study population was Medicare fee-for-service inpatients hospitalized at participating hospitals in Pennsylvania between 10/1/1998 and 9/30/1999. Inclusion criteria included having a primary ICD-9 diagnosis of pneumonia (480.0–483.99; 485–487.0), or a primary diagnosis of respiratory failure (518.81) or sepsis (038. XX) with a secondary diagnosis of pneumonia. Only the first qualifying discharge was considered for each patient.

Among the 204 hospitals functioning in PA during the study period, 101 agreed to participate in this study. For each hospital, a random sample of up to 60 discharges with qualifying ICD-9 codes was selected. For hospitals with fewer than 60 qualifying discharges, all charts were selected. In most cases, chart review data was collected by trained record abstractors either on site from the original record, or from photocopies sent to the offices of the Quality Improvement Organization. In two cases, data were collected by the hospital's own staff using an approved QIO data collection instrument (n = 2).

Patients were excluded if they had no working diagnosis of pneumonia on admission or received care limited to comfort measures, left the hospital "against medical advice", or were transferred from another acute care hospital. Patients whose race was not white or black were also excluded.

### Data abstraction

Chart review data included demographics, comorbid conditions, physical exam findings, laboratory data, and chest radiograph information. In addition, data on important processes of care for patients hospitalized with CAP were obtained by chart abstraction. These processes of care included: first antibiotics within 8 hours of admission, collection of blood cultures prior to antibiotic administration, oxygen saturation measurement within 24 hours of presentation, and concordance of antibiotic therapy with national guidelines. [7]

After initial training the abstractors performed data collection on charts were assessed using gold-standard cases that had been previously evaluated by multiple expert abstractors. If the abstractors did not achieve 95% accuracy, they underwent further training until they had an error rate of less than 5%. In addition, 10% of charts were reabstracted during the review process to monitor the accuracy of chart review. The error rate for these reabstracted charts remained less than 5%.

### Risk adjustment

The pneumonia severity index (PSI) was used to assess severity of illness at presentation [14] The PSI is a validated prediction rule for 30-day mortality in patients with CAP. Patients are classified into one of five risk classes based on three demographic characteristics, five comorbid illnesses, five physical examination findings, and seven laboratory and radiographic findings at the time of presentation. The PSI was developed and validated using data from a large prospective cohort study, in which 30-day mortality ranged from 0.1% for Class I to 27% for Class V for patients. [14]

### Sampling

Due to the relatively small number of black patients in the cohort (n = 240) we performed a modified resampling procedure of the white cohort with matching to the black patients on age and gender [15] We included all black patients in the study sample, and performed multiple resampling of three white patients matched for age (< 65, 65–74, 75–84, and ≥ 85) and gender to each black patient in the sample. Matching was used to filter out demographic imbalances between the populations. This resampling was performed 10 times and the results were pooled for analysis.

### Statistical analyses

Univariate statistics were used to compare sociodemographic and clinical characteristics between white and blacks patients. Categorical variables were analyzed using the Chi-square test and continuous variables were analyzed using Student's t-test.

Separate discrete conditional logistic regression models were estimated for each of the individual process of care measures, and for 30-day mortality [16] The PSI score and race were entered as independent variables into the models. In addition we assessed the significance of any clinical variables not included in the PSI and significant at P < 0.10 into regression models using a step-wise forward method. However none of these additional variables were significant so they were excluded from the models. Interactions terms were assessed for each of the models however none were statistically significant so they were not included in any of the models.

## Results

Of 4889 charts requested, the complete medical record was available for 4823. Of these, 4034 patients, 240 of whom were black, were eligible for inclusion in the study. Patients were excluded because they were neither white or black (N = 231) or because they had no working diagnosis of pneumonia on admission (n = 413), their care was restricted to comfort measures (n = 173), they were transferred from another acute care facility (n = 37), or they left "against medical advice" (n = 14).

For each of the ten resamplings 720 white patients were sampled and matched to the 240 black patients based upon the age and gender as previously discussed. The clinical and demographic characteristics of the study population are presented in Table 1. For our analysis of racial differences in care, the age and gender distribution of the whites was similar to that of the blacks because of our matching strategy. However, blacks continued to have higher PSI scores, indicating greater severity of illness, as well as more commonly having each of the comorbid conditions (malignancy, chronic renal disease, liver disease, congestive heart failure and history of stroke) that contribute to the PSI. There were no other statistically significant differences between the two groups.

In univariate analysis mortality at 30-days was 7.8% for whites and 5.8% for blacks (p = 0.3), and 82.1% of whites received antibiotics within 8 hours as compared to 75.7% of blacks (p = 0.04). Regarding blood culture performance, 96.4% of white and 97.1% of blacks had blood cultures obtained within 24 hours, and 84.8% of whites and 77.8% of blacks had blood cultures obtained prior to antibiotics (p = 0.03). Oxygenation saturation was assessed within 4 hours of 88.9% of whites and 93.9% of blacks (p = 0.03).

**Figure 1:**
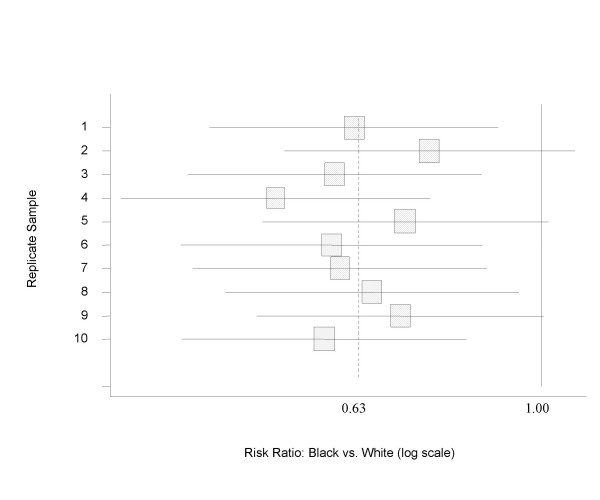
**Results of regression analysis for race with the dependent variable initial antibiotics within 8 hours. **Forest plot demonstrating the results of the 10 individual conditional logistic regressions models for the dependent variable initial antibiotics within 8 hours after adjusting for severity of illness using the PSI. The presented relative risk with 95% confidence intervals is for the independent variable race (dichotomized into black versus white).

Figures 1 through 5 are forest plots that demonstrate the effect of race on the dependent variables. These plots show each of the 10 samplings and the results of the pooled analysis. These figures demonstrate the significant variability between the random samples for the different dependent measures.

In the regression models, after adjusting for severity of illness with the PSI, black patients were significantly less likely to receive antibiotics within 8 hours with an odds ratio (OR) of 0.63 and 95% confidence interval (CI) of 0.41 to 0.97. Black patients also had a trend towards decreased all-cause mortality at 30-day with an OR of 0.4 and 95% CI of 0.16 to 1.0. There were no significant differences between whites and blacks in regards to obtaining blood cultures prior to antibiotics (OR 0.69, 95% CI 0.32–1.47), oxygenation assessment within 24 hours (OR 1.61, 95% CI 0.85–3.04), or use of guideline concordant antibiotics (OR 0.86, 95% CI 0.62-1.71).

## Discussion

This study found significant racial differences in an important process of care for patients with CAP, specifically time to antibiotic administration. Our results support the previous studies of racial variation in pneumonia care which demonstrated racial variations in care for patients hospitalized with community-acquired pneumonia. [10-13]

Our study also suggests that these variations may have clinically important outcomes since the process measures used to assess quality of care in this study have been previously associated with increased 30-day mortality. [7-9]

Our study is also consistent with previous studies which found that blacks hospitalized with CAP have lower short-term mortality rates as compared to do whites [5,6] It is unclear why this would be the case. Possible explanations include confounders that we were not able to control for, or other important factors, which were not examined that may significantly vary by race such as sociodemographic characteristics or differences in immune response.

Racial variations in CAP are important to assess since unlike coronary artery bypass surgery, hemodialysis, and many other conditions that have been studied, the inpatient treatment of CAP is largely outside of the control of the patient. Although the patient has input into being admitted to the hospital, after that point the patient has little input into the processes of care such as choice and timing of antibiotics, diagnostic testing or location of care. This has several advantages in studies where researchers seek to determine if racial differences in care reflect patient preferences, provider decisions or some negotiation between them.

There are several possible explanations for our findings of racial variations in these processes of care. Besides the obvious conclusion that there may be biases that affect care there are several other possible factors that may be responsible that we are not able to examine. One possible factor is that there are geographic or other factors that results in blacks presenting for admission at hospitals with overall lower quality of care for patients with CAP. Although we were not able to control for this factor other studies have suggested that the reverse is usually true. [11,13] That is, minorities are more likely than whites to receive their care at tertiary teaching hospitals, which on average provide superior care as compared to other hospitals. [12,17]

**Figure 2:**
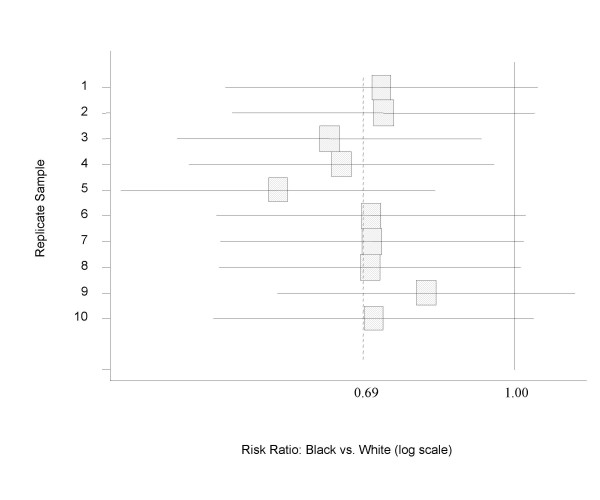
**Results of regression analysis for race with the dependent variable obtaining blood cultures prior to initial antibiotic dose. **Forest plot demonstrating the results of the 10 individual conditional logistic regressions models for the dependent variable blood cultures obtained prior to initial antibiotic dose after adjusting for severity of illness using the PSI. The presented relative risk with 95% confidence intervals is for the independent variable race (dichotomized into black versus white).

**Figure 3:**
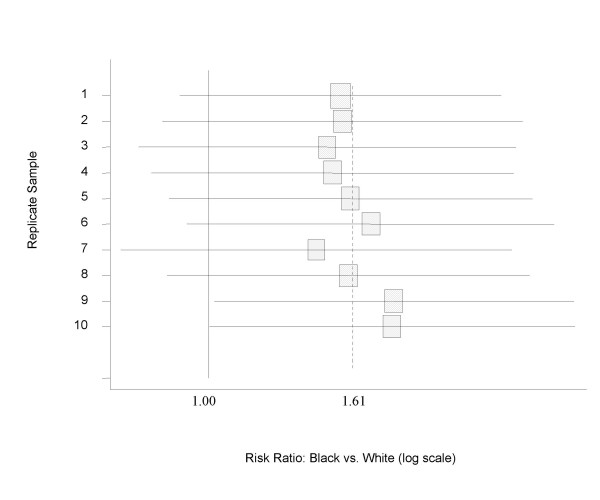
**Results of regression analysis for race with the dependent variable assessment of oxygen saturation within 24 hours. **Forest plot demonstrating the results of the 10 individual conditional logistic regressions models for the dependent variable assessing oxygen saturation within 24 hours after adjusting for severity of illness using the PSI. The presented relative risk with 95% confidence intervals is for the independent variable race (dichotomized into black versus white).

**Figure 4:**
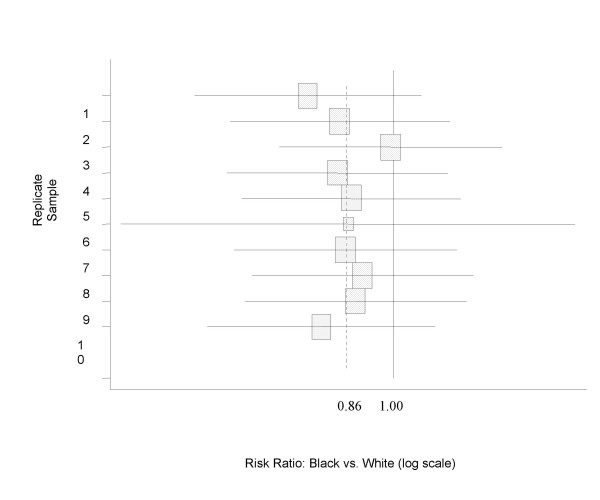
**Results of regression analysis for race with the dependent variable use of guideline-concordant antibiotics. **Forest plot demonstrating the results of the 10 individual conditional logistic regressions models for the dependent variable use of guideline-concordant antibiotics after adjusting for severity of illness using the PSI. The presented relative risk with 95% confidence intervals is for the independent variable race (dichotomized into black versus white).

**Figure 5:**
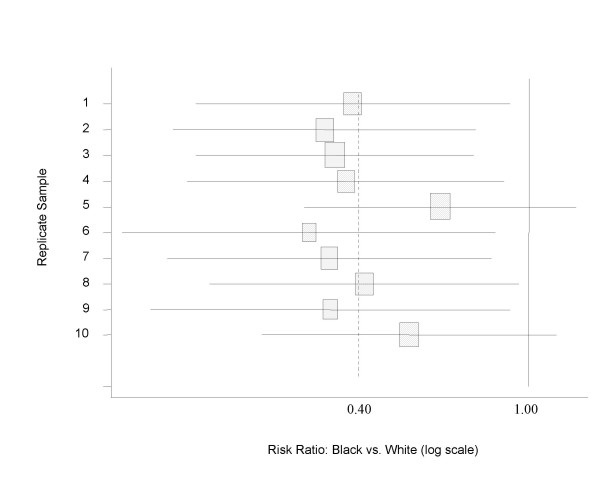
**Results of regression analysis for race with the dependent variable mortality at 30-days. **Forest plot demonstrating the results of the 10 individual conditional logistic regressions models for the dependent variable all-cause mortality at 30-days after adjusting for severity of illness using the PSI. The presented relative risk with 95% confidence intervals is for the independent variable race (dichotomized into black versus white).

To attempt to adjust for imbalances between black and white patients we used a modified resampling technique to generate 10 samples of white patients, which were matched to the black population. We then pooled these results over the 10 samples. This approach allowed us to obtain a more robust estimate of the effect racial variation may have on mortality and processes of care then would be obtained from a single random matched sample [15] Interestingly it also demonstrates the potential biases that may be present if only a single sampling is performed for a matched analysis. The forest plots (figures 1,2,3,4,5) demonstrate that for some individual samples obtained significantly different results than the pooled analysis. We feel that this technique strengthens our demonstrated results.

There are several limitations that should be acknowledged. First we did not have information on the physicians, hospitals, or the geographic locations of the providers so we were not able to adjust for clustering. Second our study was limited to Medicare patients hospitalized in Pennsylvania. It will also be important to examine whether patients with other types of insurance, such as Medicaid and managed care, and from other states have similar outcomes. In addition we were also unable to assess the robustness of our analysis using traditional techniques such as model cross validation on a new independent sample, or by randomly subdividing our current sample into a training and test samples, due to our small sample size. However we do have quasi-replication, at least in the white sample, by the multiple sampling that we performed. Finally we were unable to adjust for potential bias in pulse oximetry since this is a retrospective study. However recent work [18,19] questions the idea that pulse oximetry does not perform as well in those with increase pigmentation as compared to those with lighter pigmentation. Therefore we feel that it is unlikely that this would systematically bias the results of our study.

## Conclusions

Despite these limitations, we believe our results, and the results of other studies, allow us to conclude that blacks are less likely than whites to have processes of care that are considered to represent superior quality of CAP care. Despite this, blacks with CAP have similar, and perhaps lower mortality than whites. Further research should both investigate how to make sure all patients receive optimal CAP care, and identify the factors responsible for the paradoxical advantage in survival that is seen among blacks.

## Competing interests

None declared.

## Authors' contributions

EM and JW conceived the study. EM and JC were responsible for the analysis. EM was responsible for the initial draft of the manuscript. All authors read and approved the final manuscript.

## Pre-publication history

The pre-publication history for this paper can be accessed here:



## Figures and Tables

**Table 1:** Univariate characteristics of patients with community-acquired pneumonia by race

	**Number and % of Patients**				
		
	White		Black		
**Variable**	n = 720*	%	n = 240	%	**p-value**
**Age**					1.00
<65	132	18.3	44	18.3	
65–74	189	26.3	63	26.3	
75–84	255	35.4	85	35.4	
>84	144	20.0	48	20.0	
**Gender**					
Male	312	43.3	104	43.3	
Female	408	56.6	136	56.7	
**Length of Stay**					
1–2 days	63	8.8	23	9.6	0.663
3–6 days	375	52.2	116	48.3	
7–13 days	220	30.6	76	31.7	
>13 days	60	8.4	25	10.4	
Nursing home resident	148	21.0	51	22.2	0.774
**Pneumonia Severity Index**					0.001
I-III	280	39.0	68	28.8	
IV	296	41.2	97	41.1	
V	142	19.8	71	30.1	
**Comorbid conditions**					
Malignancy	57	8.0	28	12.2	0.073
Chronic renal disease	75	10.6	58	25.2	0.000
Liver disease	9	1.3	5	2.2	0.039
Congestive heart failure	199	20.0	80	34.9	0.057
History of stroke	105	14.9	55	23.9	0.002

*The average frequency over the 10 random samples
